# Fusion Models for Generalized Classification of Multi-Axial Human Movement: Validation in Sport Performance

**DOI:** 10.3390/s21248409

**Published:** 2021-12-16

**Authors:** Rajesh Amerineni, Lalit Gupta, Nathan Steadman, Keshwyn Annauth, Charles Burr, Samuel Wilson, Payam Barnaghi, Ravi Vaidyanathan

**Affiliations:** 1 Department of Electrical Engineering, Southern Illinois University, Carbondale, IL 62901, USA; rajeshamerineni@siu.edu (R.A.); lgupta@siu.edu (L.G.); 2 Department of Mechanical Engineering, Imperial College London, London SW7 2AZ, UK; n.steadman17@imperial.ac.uk (N.S.); keshwyn.annauth.20@ucl.ac.uk (K.A.); charles@thecornerapp.com (C.B.); s.wilson@sergtechnologies.com (S.W.); 3 Athletec Inc., Manchester M17 1QR, UK; 4 Serg Technologies Inc., London SW7 2LQ, UK; 5 Department of Brain Sciences, Imperial College London, London W12 0NN, UK; p.barnaghi@imperial.ac.uk; 6 Dementia Research Institute Care Research and Technology Centre (DRI-CR&T), London W12 0BZ, UK

**Keywords:** sports biomechanics, human performance, motion tracking, wearable sensors, IMUs, sensor fusion, DTW, CNNs, deep learning

## Abstract

We introduce a set of input models for fusing information from ensembles of wearable sensors supporting human performance and telemedicine. Veracity is demonstrated in action classification related to sport, specifically strikes in boxing and taekwondo. Four input models, formulated to be compatible with a broad range of classifiers, are introduced and two diverse classifiers, dynamic time warping (DTW) and convolutional neural networks (CNNs) are implemented in conjunction with the input models. Seven classification models fusing information at the input-level, output-level, and a combination of both are formulated. Action classification for 18 boxing punches and 24 taekwondo kicks demonstrate our fusion classifiers outperform the best DTW and CNN uni-axial classifiers. Furthermore, although DTW is ostensibly an ideal choice for human movements experiencing non-linear variations, our results demonstrate deep learning fusion classifiers outperform DTW. This is a novel finding given that CNNs are normally designed for multi-dimensional data and do not specifically compensate for non-linear variations within signal classes. The generalized formulation enables subject-specific movement classification in a feature-blind fashion with trivial computational expense for trained CNNs. A commercial boxing system, ‘Corner’, has been produced for real-world mass-market use based on this investigation providing a basis for future telemedicine translation.

## 1. Introduction

Mechatronic systems recognizing human activity are now fundamental components in biophysical analysis, with strong impact in fields such as physiotherapy, telemedicine, smart homes, rehabilitation, human-robot interface, and athletics (e.g., [1,2,3,4,5,6,7,8]). A wide range of activity-aware systems including smart phone apps (e.g., Galaxy Moves App, iPhone Moves App, iPhone Health Mate App, iPhone Fitbit App), athletic wearables (e.g., Nike Fuelband, Jawbone UP24, Fitbit Flex, Fitbit One, Fitbit Zip, Digi-Walker SW-200) and fall detection devices (e.g., Philips Lifeline, Lively Mobile, Sense4Care, Angel4) are commercially available today. Despite this range, most wearables remain limited to simple metrics such as step count, heart rate, and calories expended [9]. Though initial sales are promising, a staggering 1/3 of users abandon wearable devices [10], speaking to obvious challenges in transience and sustainability.

There is a significant need for systems that go beyond base movement metrics. Specific action classification and performance feedback on extremity movement in real-time [4,6,9,10] is in very high demand. In controlled or prepared environments, such information can be obtained with optical/camera systems, (e.g., ViconTM-Vicon, Denver, CO, USA) which have the benefit of high accuracy, but are also costly, non-portable, vulnerable to camera occlusion, and are challenging in the field. A smaller body of work has addressed these limitations with wearable vision systems (surveyed in [8]), however a robust compact system capable of tracking limb movements in the field has not been realized for large sets of classification problems. Generalizability to new or individual classes of movements without manual or bespoke feature extraction is critical for transition between types of action classification and personalized performance assessment.

Smaller systems such as inertial measurement units (IMUs) surmount issues of portability, occlusion, and price, yet demand signal fusion to provide information on movement beyond simple metrics such as step count. Recent innovation has surmounted this gap with learning algorithms fusing inertial data in specific applications. Examples include: treatment of neural dysfunction such as stroke [1] or Parkinson’s Disease [11], motion recognition in smart homes [5], athletic training parameterized for specific sports [3], and artificial limb/robotic control [7]. Despite these advances, translation for widespread use demands less reliance on specific features of movement in one arena. Complex recognition problems and individual variance in movement classes must be addressed [5]. Activity recognition with no reliance on ‘hand engineered’ feature identification is challenging due large variability in motor movements for a given action. This necessitates broader classes of learning [12] and the capacity to fuse ensembles of individualized heterogeneous data [3,5,6,9,13]. Sensors, embedded systems, and cloud connectivity have evolved the field from a ‘device’ to a ‘systems’ perspective [9]; algorithms, hardware and IoT are fundamentally coupled, and must be treated as an integrated whole. Finally, real-world use demands algorithms be computationally efficient enough for real-time use, ideally as embedded systems on low-power edge devices (e.g., wearables) that may function through communication gaps in the field [14].

## 2. Materials and Methods

### 2.1. Scope of Work

In this investigation, we introduce a set of models to fuse information from wearable sensors to learn broad classes of human movement without dependency of any assessed features of that movement. The models are formulated generically, then validated in two sport applications. We have selected dynamic time warping (DTW) and convolution neural network (CNN) classifiers for the development of movement classification systems. The primary reason for selecting these two methods is that the multi-axial signals can be fed directly into our fusion classifiers without having to extract “hand-engineered” features. Consequently, DTW and CNN classifiers do not suffer the main drawback of classifiers that use hand-engineered features whose performance is highly dependent on the choice of the extracted features. Moreover, the selection of a set of features for a given problem is more of an art than science.

It is well known that human movements experience non-linear trial-to-trial variations which typically include expansions and/or compressions in signal segments and latency shifts in the peaks. DTW classifiers are a clear choice because they are specifically designed to handle such non-linear variations in one-dimensional signals through non-linear alignment [15,16,17,18,19,20,21,22,23,24,25,26,27,28,29]. Furthermore, the DTW classifiers can also serve as benchmark for comparing performance between classifiers. CNNs are not an obvious choice because they are primarily designed to classify two-dimensional and multidimensional data such as images in computer vision. Recent studies, however, have shown that CNNs can also be used to classify multivariate time series [30,31,32,33] and human-activity activity recognition problems using multi-modal sensors [34,35,36,37,38,39] through the generation of images [33,38] or by combining uni-axial signals into matrices [36,37,39]. However, the tolerance of CNNs to non-linear signal variations and the exploitation of coupling between uni-axial signals have not been specifically addressed in the detailed manner as described in this study.

### 2.2. Multisensor Fusion Validation Application: Combat Sport

We have chosen to test and validate our fusion classifier models in combat sport given the diversity of arm and leg movements, the fact that movements are representative of those necessitating multi-axial recognition, and the capacity to collect and test large sets of meaningful data. The use of IMUs in combat sports has grown in recent years (reviewed in [6]), though existing approaches tend to be focused on metrics or specific signal features. In general, classification systems that exploit information from ensembles of multi-axial sensors are capable of improving, quite significantly, the performance over uni-axial classifiers [40,41,42,43,44,45]. However, multi-sensor classifiers tend to be more complex because of the need to incorporate fusion methods to combine “information” from the multiple sensors. The fusion methods can be divided into “input-level fusion” and “output-level” fusion. For input-level fusion, also called early fusion, the information can be input data or features extracted from the data. The information in output-level fusion, also called late fusion, is typically the decisions of the uni-axial classifiers or some measure at the outputs of the uni-axial classifiers.

The fusion models developed in this study for classifying combat sport movement are formulated generically and then validated by classifying 24 classes of kicking movements in taekwondo and 18 classes of punching movements in boxing. To our knowledge this is the first set of generalized non-feature specific models demonstrated on such a large number of classes in either activity [6].

### 2.3. Investigation Goals

Our first goal is to introduce data input models which: (a) facilitate fusion of information at the input and output levels and (b) are generalizable for use in conjunction with a broad range of diverse classifiers. The second goal is to design DTW and CNN classifiers for human movement identification using these input models. The third goal is to design experiments to classify boxing and taekwondo strikes. The final goal is to compare the DTW and CNN-based classification systems with respect to accuracy, complexity, flexibility, and the potential to obtain further improvements in performance. We offer these findings as a basis for translation of wearables for a range of human performance and healthcare applications.

### 2.4. Orginisation of Paper

Section 3 describes the four movement classifier input models. Section 4 and Section 5 describe the DTW and CNN models that are used in conjunction with the input models. Section 6 describes data collection and the strike movements for the validation studies in combat sports. Section 7 outlines classification results for 24-class kicking and 18-class punching movements. Section 8 briefly describes translation to a commercial product as evidence of novelty and impact while Section 9 summarizes conclusions from the investigation.

## 3. Classifier Input Models

**Figure 1:**
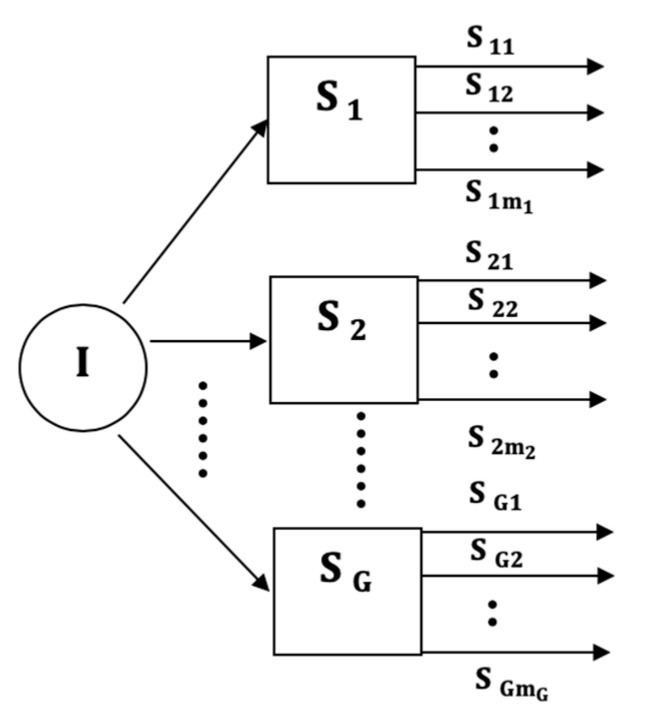
The Vector Input (VI) model.

**Figure 2:**
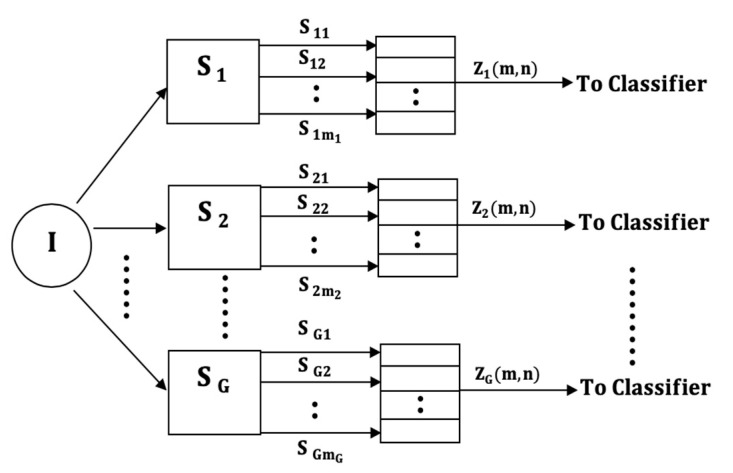
The Local Matrix Input (LMI) model.

**Figure 3:**
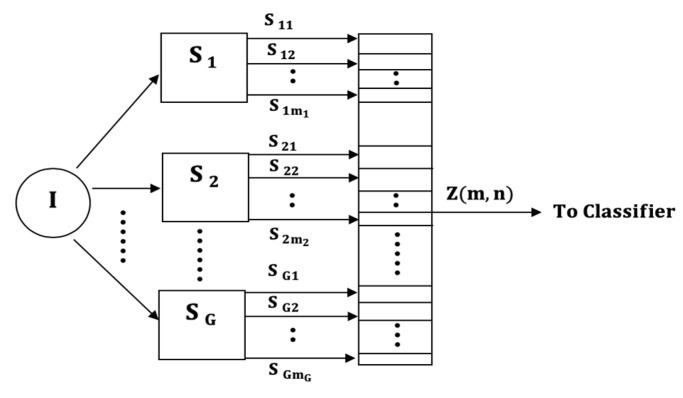
The Global Matrix Input (GMI) model.

**Figure 4:**
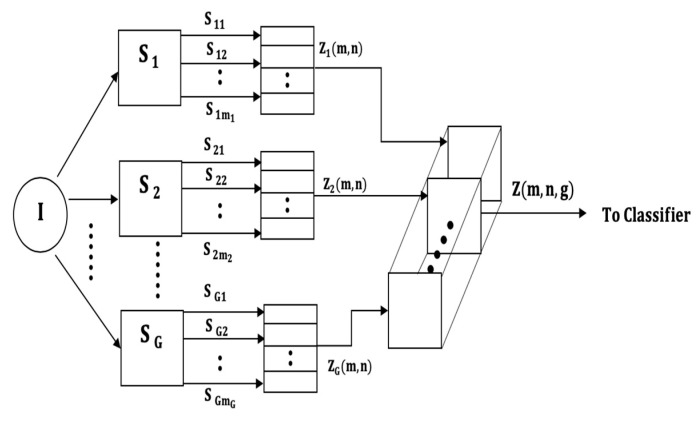
The Global Cuboid Input (GCI) model.

We propose four input models, which differ in the way the multi-axial sensor signals are presented as inputs into the subsequent classification stages. The four classifier input arrangements are summarized in Figure 1, Figure 2, Figure 3 and Figure 4. The models can be contrasted by noting the level of fusion incorporated in the models. In the formulations of the classification models, a movement is represented by I and it is assumed that the movement belongs to one of H movement classes, $\omega_{h},\;h=1,2,\ldots ,H$. The models are assumed to have G multi-axial sensors represented by $S_{g},\;g=1,2,\ldots ,G$, and an output of sensor $S_{g}$ is represented by $S_{gm},\;m=1,2,\ldots ,m_{g}$, where, $m_{g}$ is the number of multi-axial outputs. The term “non-linear variations” will be used to encompass latency shifts (shifts in peak positions) and expansions/compressions in signal segments.

### 3.1. Vector Input (VI) Model

The VI model, shown in Figure 1, is straightforward because it does not involve any form of input-level fusion. The figure simply shows the labeling of the sensors and the sensor outputs. This model is suitable for systems that classify each uniaxial signal $s_{gm}$ independently. The number of independent classifiers in such a system, therefore, is $M_{G}=\sum_{g=1}^{G}m_{g}$. Systems using this input model need fusion at the output level to determine the class of the movement signal. Of the four input models, the VI model is the most versatile because the sensors can be heterogeneous and can have a different number of axes. Furthermore, the sensor outputs can have different durations and do not have be synchronized with respect to non-linear variations. However, the resulting classifiers are the most complex because they require a classifier for each multi-axial signal and output-level fusion to combine the information from the $M_{G}$ classifier outputs in order to determine the input class.

### 3.2. Local Matrix Input (LMI) Model

The LMI model is designed for systems that classify the uniaxial outputs of each sensor separately by fusing the signals of each sensor into a matrix as shown in Figure 2. That is, the outputs of each multi-axial sensor $S_{g}$ are fused into a local intra-sensor matrix

(1) $$Z_{g}\left(m,n\right),\;m=1,2,\ldots ,m_{g};n=1,2,\ldots ,n_{g}$$

where, $n_{g}$ is the duration of the outputs of sensor $S_{g}$ (assumed equal in each sensor). The number of matrices is equal to the number of sensors G. The intra-sensor matrix to classify the signals of sensor $S_{g}$ can be written as

(2) $$Z_{g}\left(m,n\right)=\nabla_{m=1}^{m_{g}}s_{gm},\;g=1,2,\ldots ,G$$

where, the fusion operation is represented by ∇. Each matrix can be classified independently, and some form of output-level fusion can be applied to determine the class of the movement signal. The resulting classification system, therefore, is a hybrid system which includes both input and output-level fusion. This LMI input model is more restrictive than the previous model because the multi-axial sensor outputs must the same durations within each sensor (not across all sensors) in order to fuse them into a matrix. Moreover, the multi-axial sensor outputs are assumed to experience synchronized non-linear variations within each sensor. The advantage of the LMI model is that the number of classifiers is reduced to G when compared with the $M_{G}$ classifiers needed in the previous VI model.

### 3.3. Global Matrix Input (GMI) Input Model

The third model, involving only input-level fusion in the classifiers, is designed to classify the uniaxial sensor signals of all sensors by fusing the signals into a global inter-sensor matrix shown in Figure 3. The inter-sensor matrix is formed by fusing all multi-axial outputs into a matrix $Z\left(m,n\right),\;m=1,2,\ldots ,M_{G};\;n=1,2,\ldots ,N$, where, N is the duration of each sensor output (assumed equal). That is, each row of Z(m,n) is an output of a multi-axial sensor. The global input matrix is, therefore, given by

(3) $$Z\left(m,n\right)=\nabla_{g=1}^{G}\nabla_{m=1}^{m_{g}}s_{gm}$$

This fusion operation is equivalent to fusing the LMI matrices into a matrix, therefore, the global matrix can also be written as

(4) $$Z\left(m,n\right)=\nabla_{g=1}^{G}Z_{g}\left(m,n\right)$$

Unlike the two previous models, classifiers using this input model do not require output-level fusion because only a single classifier is needed to classify the global matrix. However, it is important to note that the resulting classifier is more restrictive than the two previous models because the following assumptions are made:

1. The multi-axial outputs have the same durations within and across all sensors in order to fuse them into a global matrix.
2. The multi-axial sensor outputs experience synchronized non-linear variations within and across all sensors.

### 3.4. Global Cuboid Input (GMI) Input Model

If the number of uniaxial outputs and the output durations of all G sensors are assumed equal, the LMI matrices can also be fused in a cuboid which can be represented by

$$Z\left(m,n,g\right)=\Delta_{g=1}^{G}Z_{g}\left(m,n\right),\;m=1,2,\ldots ,M;n=1,2,\ldots ,N;g=1,2,\ldots ,G$$

where Δ is the cuboid fusion operation, M is the number of uniaxial outputs of each sensor and N is the duration of each uniaxial signal. This input model, shown in Figure 4, is the most restrictive because it requires an additional condition to be met, viz., the sensors must have an equal number of uniaxial outputs.

In summary, there is a trade-off between classifier complexity and flexibility using the four input models. A suitable way to overcome the equal duration restriction is to duration normalize the signals to have a common length through linear expansion or compression. For example, the common length can be chosen to be the average duration of all sensor outputs for the GMI model and the average of outputs of each sensor for the LMI model. However, there is no simple way to overcome the non-linearity restriction and the performance of the GMI and LMI models can be expected to drop if this assumption is violated. The GCI model cannot be implemented if the number of outputs across all sensors is not equal. Another issue to take into account is the similarity of the sensors. If the sensors are heterogeneous, the heterogeneous signal amplitudes have to be normalized, for example, using min-max normalization. Even if the sensors are homogeneous, normalization within each sensor is also required to account for the varying ranges of the uni-axial signal amplitudes.

The sections that follow present the formulations of three DTW based models and the four CNN based models to classify multi-axial multiple-sensor movement signals using the four input models. It can be shown that for the DTW implementations, the GCI model is equivalent to GMI model, therefore, the GCI model is not implemented. A DTW classifier is explicitly split into two operations: discrepancy computation and a decision rule. The discrepancy computation operation determines the dissimilarity score between the aligned test and reference signals of movements and the decision rule uses these discrepancy scores to assign the test movement into one of the movement classes.

## 4. Dynamic Time Warping (DTW) Classifier

DTW has been applied in numerous applications to measure the dissimilarity between pairs of sequences that experience non-linear variations in the segments of the sequences. A sample of applications employing DTW include speech recognition [19,23], shape recognition [15,16,25], clustering [17,24], gene expression [26], financial time series matching [29], and classifying human actions in sports [27]. In order to facilitate the understanding of the formulations of the three DTW-based fusion models, a brief description of one, two, and three-dimensional DTW algorithms follows next.

### 4.1. Dynamic Time Warping (DTW) Algorithms

Given a pair of signals X and Y and a local cost function w(k) to reflect the discrepancy between the elements of X and Y, the goal of DTW is to determine an alignment function W={w(1),w(2),…,w(K)}, such that the overall normalized cost

(5) $$A_{XY}=\left(1/K\right)\overset{K-1}{\underset{k=0}{\sum }}d\left[w\left(k\right)\right]$$

is minimized subject to a set of end-point, monotonicity, and continuity constraints. $A_{XY}$ is a measure of the discrepancy between signals X and Y after optimal alignment. The most often used cost functions include the Euclidean, Manhattan, and Euclidean-squared distance metrics. Dynamic programming is used to solve the optimization problem.

The steps to align one, two, and three-dimensional signals are quite similar except for the computation of the local cost function. For example, if the Euclidean distance is used, the cost function for the one-dimensional (vector) DTW algorithm is

(6) d[w(k)]=||X(i(k))−Y(j(k)||.

For the two-dimensional (matrix) case, the cost function is given by

(7) d[w(k)]=||X(:,i(k))−Y(:,j(k))||

where, the notation Z(:,t) is used to denote column t of a matrix Z. Note that the number of rows in the two matrices must be equal but the number of columns can be different. Similarly, the cost function for the three-dimensional (cuboid) extension is given by

(8) d[w(k)]=||X(:,i(k),:)−Y(:,j(k),:)||

where, the notation Z(:,t,:) is used to denote a depth-frame t of a cuboid Z. For this case, the number of rows and depth of the two cuboids must be equal but the number of columns can be different. Also note that cuboid alignment can also be implemented as matrix alignment by fusing the height-width frames into an augmented matrix because the resulting column-to-column cost function is equal to the frame-to-frame cost function. However, the matrix alignment cannot be implemented as cubic alignment if the number of rows in the frames are unequal. In this study which involves the classification of sensor signals arranged as vectors, matrices, and cuboids, the corresponding DTW classifiers will be referred to as V-DTW, M-DTW, and C-DTW, respectively.

In order to design a DTW classifier for a given problem, a reference template for each pattern is typically estimated from the signals in their respective training sets. The sample mean vector is used often because it best represents the signals in the training set in the sense of minimizing the sum of squared distances from itself to the signals in the training set. However, this does not necessarily imply that the sample mean is the best template choice for a particular problem. Modified averaging procedures which take non-linear variations into account have been proposed to generate templates that can be used in DTW algorithms [18]. In fact, other measures of central tendency (C-T) such as the median, Winsorized mean, trimmed mean, and tri-mean can also be used. What is important to note is that a better C-T estimate does not necessarily result in a better template for classification problems. Therefore, attempting to predict which C-T estimate will yield the best template for a given problem is not easy and the selection of a template is usually determined through trial-and-error.

### 4.2. DTW Implementation of VI Model (DTW-1)

**Figure 5:**
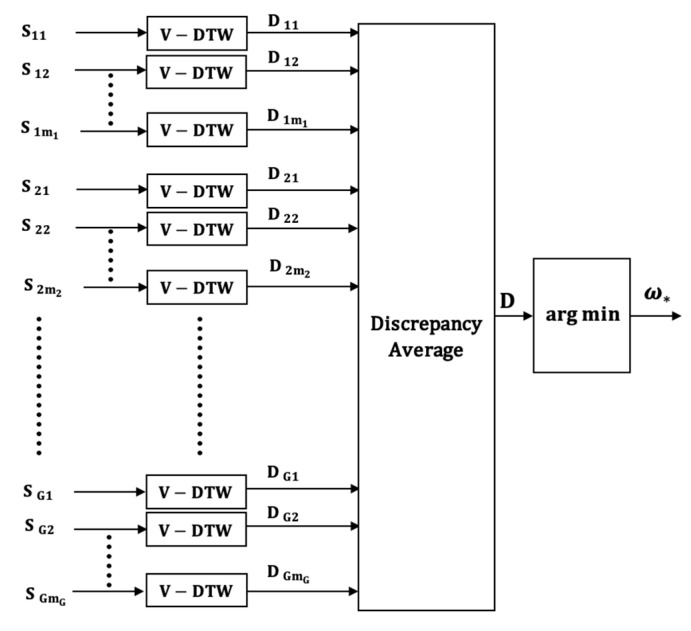
The DTW-1 classification model.

The DTW based classification model which uses the VI model is illustrated in Figure 5. The model, referred to as DTW-1, consists of one independent V-DTW classifier for each multi-axial sensor output. Therefore, the number of V-DTW classifiers is $M_{G}$. The discrepancy scores of the $M_{G}$ classifiers are fused through averaging in order to determine the class of the impact signal.

*Discrepancy computation*: The output of the V-DTW operator for the uni-axial signal $s_{gm}\;$is the discrepancy score vector $D_{gm}=\left(d_{gm}^{\omega_{1}},\;d_{gm}^{\omega_{2}},\ldots ,d_{gm}^{\omega_{H}}\right)$, where, $d_{gm}^{\omega_{h}}$ is the discrepancy between a test sequence $s_{gm}^{T}$ and the reference sequence $s_{gm}^{h}$.

*Output Fusion Rule*: The discrepancy scores of the $M_{G}$ V-DTW operators are averaged and the resulting averaged discrepancy fusion vector is given by

$$D=\left(D^{\omega_{1}},\;D^{\omega_{2}},\;\ldots ,D^{\omega_{H}}\right)$$

where:

(9) $$D^{\omega_{h}}=\left(\frac{1}{M_{G}}\right)\left[\overset{m_{1}}{\underset{m=1}{\sum }}d_{1m}^{\omega_{h}}+\overset{m_{2}}{\underset{m=1}{\sum }}d_{2m}^{\omega_{h}}+\cdots +\overset{m_{G}}{\underset{m=1}{\sum }}d_{Gm}^{\omega_{h}}\right].$$

*Decision Rule*: The test movement $I_{T}$ is assigned to the class that yields the least discrepancy using the following rule:

(10) $$\omega_{*}=\arg\;\min\left[\;D^{\omega_{h}}\right],\;h=1,2,\ldots ,H.$$

### 4.3. DTW Implementation of the LMI Model (DTW-2)

**Figure 6:**
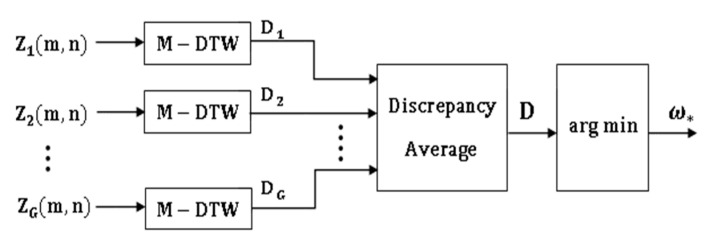
The DTW-2 classification model.

The use of M-DWT in conjunction with the LMI model is illustrated in Figure 6. In this hybrid input and output-level fusion approach, each intra-sensor matrix is classified independently using M-DWT and the class of the movement is determined by averaging the discrepancy scores of each classifier.

*Local Discrepancy Computation*: The system has one M-DTW classifier for the outputs of each multi-axial sensor. For the M-DTW classifier for sensor $S_{g}$, let $Z_{g,T}\left(m,n\right)$ and $Z_{g,h}\left(m,n\right)\;$be the local input matrix of a test movement and a reference movement of class h, respectively, and let the output of the M-DTW operator be the discrepancy vector $D_{g}=\left(d_{g}^{\omega_{1}},d_{g}^{\omega_{2}},\;\ldots ,d_{g}^{\omega_{H}}\right)$. The element $d_{g}^{\omega_{h}}$ is the discrepancy score between $Z_{g,T}\left(m,n\right)$ and $Z_{g,h}\left(m,n\right)$.

*Output Fusion Rule:* For this case, the outputs (discrepancy scores) of the G DTW operators are fused using an averaging operation. The averaged discrepancy fusion vector is given by

(11) $$D=\left(D^{\omega_{1}},\;D^{\omega_{2}},\;\ldots ,D^{\omega_{H}}\right)$$

where,

(12) $$D^{\omega_{h}}=\left(\frac{1}{G}\right)\overset{G}{\underset{g=1}{\sum }}d_{g}^{\omega_{h}}$$

*Decision Rule:* The test movement $I_{T}$ is assigned to the class $\omega_{*}$ using the rule in Equation (10).

### 4.4. DTW Implementation of the GMI Model (DTW-3)

**Figure 7:**
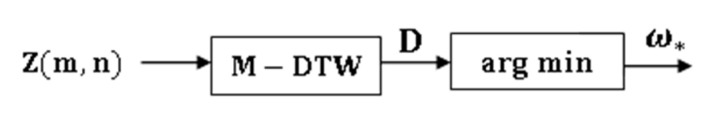
The DTW-3 classification model.

The DTW classifier that uses the GMI model is illustrated in Figure 7. In this input-level fusion approach, the system has one M-DTW classifier to classify the global inter-sensor matrix. The discrepancy scores between a test movement and reference movements are computed and the test movement is assigned to the class which yields the smallest score.

*Discrepancy Computation*: If the global input matrices of a test movement $I_{T}$ and reference template of movement $I_{h}$ are represented by $Z_{T}\left(m,n\right)$ and $Z_{h}\left(m,n\right),\;$respectively, the output of the M-DTW operator is the discrepancy vector $D=\left(D^{\omega_{1}},\;D^{\omega_{2}},\;\ldots ,D^{\omega_{H}}\right)$ in which element $D^{\omega_{h}}$ is the discrepancy score between $Z_{T}\left(m,n\right)$ and $Z_{h}\left(m,n\right)$.

*Output Fusion Rule*: none required.

*Decision Rule*: The test movement $I_{T}$ is assigned to the class $\omega_{*}$ the using the rule in Equation (10).

## 5. Convolution Neural Network (CNN) Classifiers

CNNs are a class of deep learning networks that is capable of performing well in computer vision problems such as large-scale object classification and detection in images [46,47,48,49,50,51]. One of the most striking features of CNNs when compared with other traditional classifiers, including fully connected neural networks (FCNs), is that a minimal amount of preprocessing is required to generate the input to the network. For example, an image can be processed directly without having to convert it into a vector. Converting images to vectors results in a very long input vector which can lead to the curse of dimensionality in traditional classifiers and a large network for FCNs which in turn results in a large number of network parameters and overfitting problems. Though seldom discussed, converting an image into a vector leads to a poor representation of the input image because it loses the relationship between a pixel and its vertical and diagonal neighbors which is important for local feature detection. The most often used methods to overcome the dimensionality-related problems is through feature extraction. However, as noted in the introduction, selecting a set of features for a given problem is more an art than science and features are typically selected through trial-and-error. CNNs overcome these problems by applying feature extracting filters directly to the image and most importantly, learning the filter weights through training rather than using prior knowledge to hand-engineer the weights. Moreover, the overfitting problem is reduced through parameter sharing in which the same filter is used to determine each element in the feature map.

**Figure 8:**
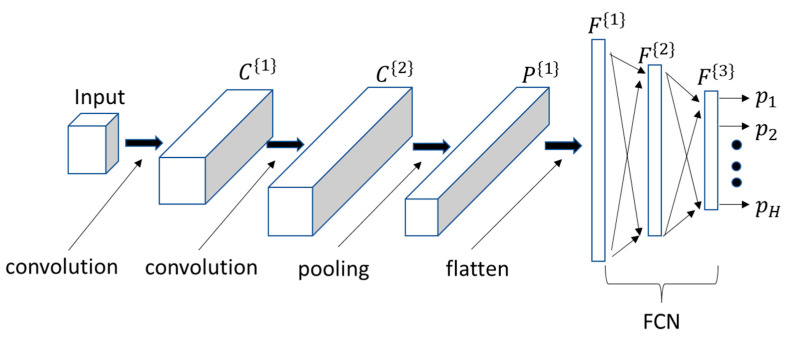
Block diagram of a CNN with two convolution layers and a pooling layer.

A typical CNN has an input layer, an output layer, and hidden layers consisting of convolution, pooling, and fully connected layers. The network architecture is defined by the number and arrangement of the convolution and pooling layers. Figure 8 is an illustration of a CNN with two convolution layers $C^{\left\{1\right\}}$ and $C^{\left\{2\right\}}$ followed by a pooling layer $P^{\left\{1\right\}}$ and a FCN with layers $F^{\left\{1\right\}}$, $F^{\left\{2\right\}}$, and $F^{\left\{3\right\}}$ (output layer). The input to the first fully connected layer is the flattened (concatenated) output from the pooling layer. In general, the dimension of a convolution layer depends on the number of convolution filters, the filter stride, and the type of convolution (valid or same). A pooling layer dimension depends on the size and stride of the pooling filters. For classification problems, the output layer is typically a softmax layer with one output for each pattern class. The network is trained using the gradient descent backpropagation algorithm.

The two notable operations performed in CNNs are convolution and pooling. Each convolution layer contains a set of filters which have spatial dimensions much smaller than those of the image, however, the depth (number of channels) is usually the same as the input. A bias is added to the filtered outputs which are then passed through a non-linear activation such as the ReLu function to yield the feature maps. The feature maps are stacked into cuboids to form the output of the convolution layer in which the number of channels is equal to the number of filters. If the convolution layer is followed by a pooling layer, the spatial dimension is reduced by subsampling blocks in each feature map in the convolution layer output. Max pooling, which replaces a block with the maximum value, is the most often used pooling operation. Pooling serves two purposes: it progressively reduces the spatial dimension thus decreasing the overfitting problem through the reduction in the number of parameters and selects the most robust features.

The actual operation that is performed in the convolution layer is correlation and not convolution. The term “convolution”, therefore, is incorrectly used. However, if the input or the filter is folded (1-d case) or rotated (2-d and 3-d cases), the correlation and convolution operations are equivalent. Therefore, it is assumed that one of the inputs has been pre-folded or pre-rotated prior to the actual correlation operation performed in the convolution layer.

The following sections describe four implementations of CNNS that use the vector, matrix, and cuboid input models. The models can be distinguished by the convolution operations in the first stage and the output-level fusion operation. In order to do so, the input and output of the first convolution layer are assumed to be generalized cuboids with dimensions $\left(H^{\left[0\right]}\times W^{\left[0\right]}\times D^{\left[0\right]}\right)$ and $\left(H^{\left[1\right]}\times W^{\left[1\right]}\times D^{\left[1\right]}\right)$, respectively. Using this notation, a d-dimensional vector and (m×n) matrix are represented as (1×d×1) and a (m×n×1) generalized cuboids, respectively. If a pooling layer follows, the output of the pooling layer is assumed to have dimensions $\left(H^{\left[1,p\right]}\times W^{\left[1,p\right]}\times D^{\left[1,p\right]}\right)$. The filters in the first convolution layer have dimensions represented by $\left(f_{h}^{\left[1\right]}\times f_{w}^{\left[1\right]}\times D^{\left[0\right]}\right)$ and the pooling filter by $\left(f_{h}^{\left[1,p\right]}\times f_{w}^{\left[1,p\right]}\times 1\right)$. The dimensions are related as follows:

(13) $$H^{\left[1\right]}=\left[1+\left(H^{\left[0\right]}-f_{h}^{\left[1\right]}+2p\right)/s_{c}\right]$$

(14) $$W^{\left[1\right]}=\left[1+\left(W^{\left[0\right]}-f_{w}^{\left[1\right]}+2p\right)/s_{c}\right]$$

(15) $$D^{\left[1\right]}=K^{\left[1\right]}$$

(16) $$H^{\left[1,p\right]}=\left[1+\left(H^{\left[1\right]}-f_{h}^{\left[1,p\right]}\right)/s_{p}\right]$$

(17) $$W^{\left[1,p\right]}=\left[1+\left(W^{\left[1\right]}-f_{w}^{\left[1,p\right]}\right)/s_{p}\right]$$

(18) $$D^{\left[1,p\right]}=D^{\left[1\right]}$$

where, p, $s_{c}$, $s_{p}$, and $K^{\left[1\right]}$ represent the zero-padding amount, convolution stride, pooling stride, and the number of filters in the first stage, respectively. Zero-padding is employed in “same convolution” to keep the input and output dimensions equal. If p=0, the output of the “valid convolution” operation has smaller dimensions than those of the input.

Just as in the development of the DTW classifiers in the previous section, the CNN classifiers are explicitly split into two operations: computation of the posterior class probabilities and a decision rule. The posterior class probabilities are computed by the CNN and the decision rule uses these probabilities to assign the test movement into one of the movement classes. Although a pooling layer may or may not follow a convolution layer, it will be assumed that a convolution layer is followed by a pooling layer for consistency in the formulations. It will also be assumed that the convolutions are “same.” The output dimensions can be easily adjusted if the convolutions are “valid.”

### 5.1. CNN Implementation of the VI Model (CNN-1)

**Figure 9:**
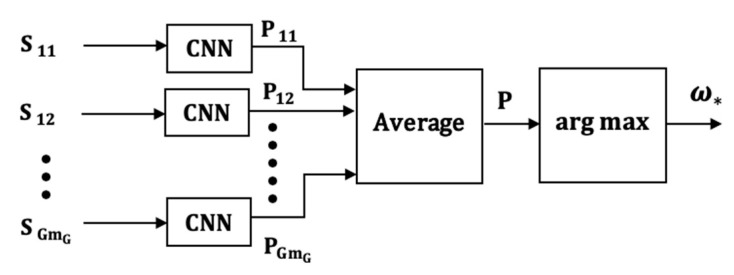
The CNN-1 classification model.

The CNN-1 classification model which uses the VI model is illustrated in Figure 9. The CNN-1 model is characterized by vector convolutions in the first layer to extract local intra-axial features and output-level fusion for combining the $M_{g}$ classifier outputs.

Because the uni-axial classifiers are identical, the CNN classifier for one uniaxial signal $s_{gm}$ is first described and the method for combining the outputs of the $M_{g}$ classifiers is described next.

*Posterior Probability Computation*: In the first convolution layer, the input vector $s_{gm}$ with generalized cuboid dimensions $\left(1\times n_{gm}\times 1\right)$ is convolved with $K^{\left[1\right]}$ filters, each with dimensions $\left(1\times f_{w}^{\left[1\right]}\times 1\right)$. Because the convolution is assumed “same”, the output ${\hat{s}}_{gm}^{\left[1,k\right]}$ of the kth filter will have the same dimensions as the input $s_{gm}$. A bias $b_{gm}^{\left[1,k\right]}$ is added to the filtered output and passed through the nonlinear ReLu activation function so that the activation of filter k in the first layer is given by

(19) $${\tilde{s}}_{gm}^{\left[1,k\right]}\left(n\right)=ReLu\left[{\hat{s}}_{gm}^{\left[1,k\right]}\left(n\right)+b_{gm}^{\left[1,k\right]}\right]$$

where, ReLu[δ]=Max[0,δ]. The output of the first convolution layer is the $K^{\left[1\right]}$ activations combined into $\left(1\times n_{gm}\times K^{\left[1\right]}\right)$ unit height cuboid represented by $S_{gm}^{\left[1\right]}$. If pooling follows and the stride and size of the pooling filter are r and (1×γ×1), respectively, the output $S_{gm}^{\left[1,p\right]}$ of the pooling layer will have dimension $\left(1\times [((n_{gm}-\gamma \right)/r)+1]\times K^{\left[1\right]})$.

In the second convolution layer, if each filter has dimension $\left(1\times f_{w}^{\left[2\right]}\times K^{\left[1\right]}\right),$ the output ${\hat{s}}_{gm}^{\left[2,k\right]}$ of the kth filter will have dimension $\left(1\times \left[((n_{gm}-\gamma \right)/r\right)+1]\times 1)$. Note that although the two functions convolved are unit height cuboids, the output is a vector. After adding a bias and passing each filtered output through the ReLu activation function, the $K^{\left[2\right]}$ activations are combined into a unit height cuboid. The width of the unit height cuboid is adjusted according to the stride if a pooling layer is added. If necessary, the convolution and pooling operations can be repeated. A flattening operation is employed to combine the rows of the last cuboid into a vector which forms the input to a fully connected feed forward neural network with $N_{gm}$ layers. Typically, the sigmoidal or tanh functions are used as activations in the intermediate hidden layers and the softmax activation is used in the output layer of the fully connected network (FCN). Cross-entropy is employed for the loss-function. Because of the softmax activation function, the outputs can be regarded as estimates of posterior probabilities given by

(20) $$p_{gm}\left(h\right)=\frac{e^{q_{h}}}{\sum_{h=1}^{H}e^{q_{h}}}\;,\;h=1,2,\ldots ,H$$

where, $q_{h}$ is the weighted sum of the inputs into a neuron h in the output layer.

The output of the CNN classifier for signal $s_{gm}$ is represented by the vector

(21) $$P_{gm}=\left(p_{gm}\left(1\right),p_{gm}\left(2\right),\ldots ,p_{gm}\left(H\right)\right);\;g=1,2,\ldots ,G,\;m=1,2,\ldots ,m_{g}$$

*Decision Rule*: The H probabilities of the $M_{G}$ CNN classifiers are averaged into a probability fusion vector represented by

(22) $$P=\left(P^{\omega_{1}},\;P^{\omega_{2}},\;\ldots ,P^{\omega_{H}}\right)$$

where,

(23) $$P^{\omega_{h}}=\left(1/M_{G}\right)\left[\overset{m_{1}}{\underset{m=1}{\sum }}p_{1m}\left(h\right)+\overset{m_{2}}{\underset{m=1}{\sum }}p_{2m}\left(h\right)+\cdots +\overset{m_{G}}{\underset{m=1}{\sum }}p_{Gm}\left(h\right)\right].$$

Using the maximum response rule, the CNN assigns the input movement to the class associated with the output that yields the largest value. That is, a test movement is assigned to class $\omega_{h}$ if

(24) $$P^{\omega_{h}}>P^{\omega_{j}},\;\mathrm{for}\;\mathrm{all}\;j\neq h$$

Equivalently, the test movement is assigned to the class given by

(25) $$\omega_{*}=\arg\;\max\left[\;P^{\omega_{h}}\right],\;h=1,2,\ldots ,H.$$

The CNN-1 model shares similarities with the Channel-Based Late Fusion models (CB-LF) described in [35,39] in the sense that there is one CNN per axis. The main difference is that the CB-LF model has one FCN and the input to the FCN is the concatenation of the features from the last convolution layer of each axis. The late fusion, therefore, is a form of inter-channel feature fusion. The CNN-1 model has one FCN for each axis and the late fusion is a form of decision fusion that occurs at the outputs of the CNNs.

### 5.2. CNN Implementation of the LAI Model (CNN-2)

**Figure 10:**
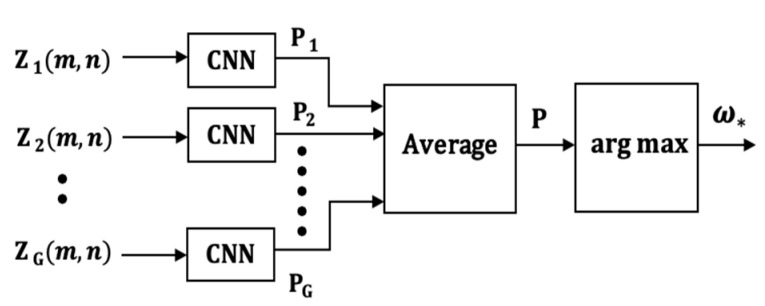
The CNN-2 classification model.

The CNN-2 classification model which uses the LMI model is illustrated in Figure 10. It is characterized by one CNN classifier for each sensor, matrix convolutions in the input layer to extract local intra-sensor features, and output-level fusion for combining the outputs of the G CNN classifiers.

*Posterior Probability Computation*: In the first layer, the sensor matrix $Z_{g}\left(m,n\right)$ with generalized dimensions $\left(m_{g}\times n_{g}\times 1\right)$ is convolved with with $K^{\left[1\right]}$ filters, each with dimensions $\left(f_{h}^{\left[1\right]}\times f_{w}^{\left[1\right]}\times 1\right)$. The output ${\hat{Z}}_{g}^{\left[1,k\right]}\left(m,n\right)$ of the kth filter is a matrix with the same dimensions as the input. A bias $b_{g}^{\left[1,k\right]}$ is added to the filtered output and passed through the nonlinear ReLu activation function. The activation of the filter, therefore, is given by

(26) $${\tilde{Z}}_{g}^{\left[1,k\right]}\left(m,n\right)=ReLu\left[{\hat{Z}}_{g}^{\left[1,k\right]}\left(m,n\right)+b_{g}^{\left[1,k\right]}\right]$$

The $K^{\left[1\right]}$ filtered outputs are combined into a $\left(m_{g}\times n_{g}\times K^{\left[1\right]}\right)$ cuboid $Z_{g}^{\left[1\right]}\left(m,n,k\right)$. If pooling follows and the stride and size of the pooling filter are r and (γ×γ×1), respectively, the output is the cuboid

$$Z_{g}^{\left[1,p\right]}\left(m,n,k\right),\;m=1,2,\ldots ,m_{g}^{\left[1,p\right]},n=1,2,\ldots ,n_{g}^{\left[1,p\right]},k=1,2\ldots ,K^{\left[1\right]})$$

where, $m_{g}^{\left[1,p\right]}=(((m_{g}-\gamma )/r)+1)$, and $n_{g}^{\left[1,p\right]}=(((n_{g}-\gamma )/r)+1)$.

In the next convolution stage, the cuboid is convolved with a cuboid filters with dimensions $\left(f_{h}^{\left[2\right]}\times f_{w}^{\left[2\right]}\times K^{\left[1\right]}\right)$. Each filtered output ${\hat{Z}}_{g}^{\left[2,k\right]}\left(m,n\right)$ resulting from the cuboid convolution is a matrix. The series of convolutions and pooling operations terminate into an FCN with a softmax output layer.

If $p_{g}\left(h\right)$ is the output of neuron h in the output layer, then, the output of classifier for matrix $Z_{g}\left(m,n\right)\;$can be represented by the vector

(27) $$P_{g}=\left(p_{g}\left(1\right),p_{g}\left(2\right),\ldots ,p_{g}\left(H\right)\right);\;g=1,2,\ldots ,G.$$

*Decision Rule*: The outputs of the G classifiers can be averaged and represented by the vector

(28) $$P=\left(P^{\omega_{1}},\;P^{\omega_{2}},\;\ldots ,P^{\omega_{H}}\right)$$

where,

(29) $$P^{\omega_{h}}=\left(\frac{1}{G}\right)\overset{G}{\underset{g=1}{\sum }}p_{g}\left(h\right).$$

A test movement is then assigned to class $\omega_{h}$ using the rule in Equation (25).

The CNN-2 model is somewhat similar to the Sensor-Based Late Fusion models (SB-LF) described in [34,39] in the sense that there is one CNN per sensor. The main difference is that the SB-LF model has one FCN and the input to the FCN is the late fusion of the features from the last convolution layer of each sensor. The CNN-2 model has one FCN for each sensor and the late fusion is a form of decision fusion that occurs at the outputs of the CNNs.

### 5.3. CNN Implementation of the GAI Model (CNN-3)

**Figure 11:**
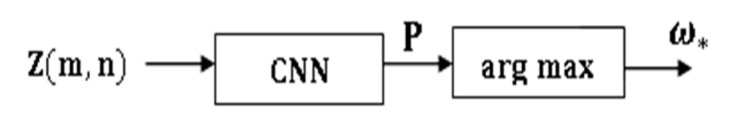
The CNN-3 classification model.

The CNN-3 classification model using the GMI model, shown in Figure 11, is characterized by matrix convolutions in the first layer to extract local intra-sensor features and no output-level fusion. A small number of inter-sensor features are also extracted from the bordering uni-axial outputs from adjacent sensors in the input matrix. The input is the global matrix Z(m,n).

*Posterior Probability Computation*: In the first layer, the matrix Z(m,n) with dimension $\left(M_{g}\times N\times 1\right)$ is convolved with with $K^{\left[1\right]}$ filters, each with dimensions $\left(f_{h}^{\left[1\right]}\times f_{w}^{\left[1\right]}\times 1\right)$. The output of the kth filter yields a matrix ${\hat{Z}}^{\left[1,k\right]}\left(m,n\right)$. A bias $b^{\left[1,k\right]}$ is added to the filtered output and passed through the nonlinear ReLu activation function. The activation of the filter, therefore, is given by

(30) $${\tilde{Z}}^{\left[1,k\right]}\left(m,n\right)=ReLu\left[{\hat{Z}}_{g}^{\left[1,k\right]}(m,n\right)+b^{\left[1,k\right]}]$$

The $K^{\left[1\right]}$ filtered outputs are combined into a $\left(M_{g}\times N\times K^{\left[1\right]}\right)$ cuboid $Z^{\left[1\right]}\left(m,n,k\right)\;$which is pooled to give the cuboid $Z^{\left[1,p\right]}\left(m,n,k\right)$. The cuboid pooling operation is not described because it is similar to the one used in the previous model. The pooled cuboid is filtered by $K^{\left[2\right]}$ cuboid filters and the output of the kth filter is a matrix represented by

(31) $${\hat{Z}}^{\left[2,k\right]}\left(m,n\right),\;m=1,2,\ldots ,M^{\left[1\right]},\;n=1,2,\ldots ,N^{\left[1\right]}$$

where, $M^{\left[1\right]}$ and $N^{\left[1\right]}$ are the height and width of the pooled output $Z^{\left[1,p\right]}\left(m,n,k\right)$. The series of convolutions and pooling operations terminate into a FCN with a softmax output layer which gives an estimate of the H movement probabilities. The softmax output is represented by the vector

(32) $$P=\left(P^{\omega_{1}},\;P^{\omega_{2}},\;\ldots ,P^{\omega_{H}}\right).$$

*Decision Rule*: a test movement is assigned to class $\omega_{h}$ using the rule in Equation (25).

The CNN-3 model is similar to the Early Fusion (EF) model described in [37,39]. The difference is mainly in the selection of the dimensions of the filters in the convolution layers.

### 5.4. CNN Implementation of the CI Model (CNN-4)

**Figure 12:**
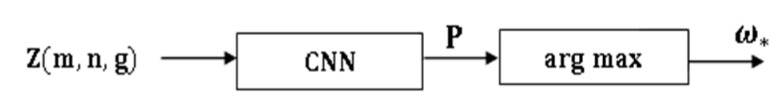
The CNN-4 classification model.

The CNN-4 classification model, shown in Figure 12, is implemented using the cuboid representation which is obtained by fusing the LMI local matrices into a cuboid. Cuboid convolutions in the first layer extract coupled intra-sensor and inter-sensor features throughout the input.

*Posterior Probability Computation*: The cuboid input Z(m,n,g) is convolved with cuboid filters $\left(f_{h}^{\left[1\right]}\times f_{w}^{\left[1\right]}\times G\right)$ and the output of the kth filter, is represented by

(33) $$Z^{\left[1,k\right]}\left(m,n\right),\;m=1,2,\ldots ,M;\;n=1,2\ldots ,N.$$

Note that convolving two cuboids with the same depth results in a matrix. The $K^{\left[1\right]}$ filtered outputs are combined into a $\left(M\times N\times K_{1}\right)$ cuboid after the biases are added and passed through the ReLu activation function. The height and width of the cuboid is adjusted if a pooling layer follows the convolution layer. Subsequent convolutions are also cuboid convolutions which result in matrices which are then combined into cuboids. An FCN with softmax outputs is implemented after the last pooling layer. The softmax output is represented by the vector

(34) $$P=\left(P^{\omega_{1}},\;P^{\omega_{2}},\;\ldots ,P^{\omega_{H}}\right).$$

*Decision Rule*: a test movement is assigned to class $\omega_{h}$ using the rule in Equation (25).

The CNN-4 model is unique because, to the best of our knowledge, there are no similar models which combine the uniaxial signals of each sensor into matrices, combine the matrices into a cuboid, and extract a combination of intra-sensor and inter-sensor features.

## 6. Experimental Data Collection

**Figure 13:**
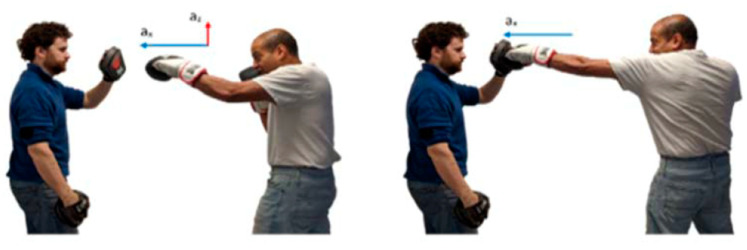
Data collection example: left jab punch (pad) (sensors under glove on wrist).

**Figure 14:**
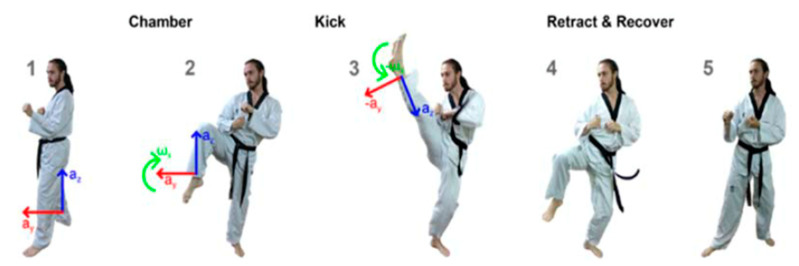
Data collection example: front right kick (sensors strapped to each ankle).

Motion capture for both taekwondo and boxing was conducted using custom Inertial Measurements Units (IMUs), developed in previous motion tracking research [7] as a basis for a commercial product. The IMU consists of a 3-axis accelerometer and 3-axis gyroscope; the ranges of the two sensor modules was set at ±16 g and ±2000 dps respectively to capture the full range of motion in both sports. Sampling frequency was constant for both sports, at 100 Hz. Data was streamed in real time from the IMU to the control computer via Bluetooth 4.0 communication. The IMU module was placed on the striking limb and held by Velcro straps. A pouch was sewn on the inside of the strap to keep IMU positioning consistent throughout the data collection process. Positioning and axis orientation of the IMU for boxing and taekwondo are outlined for sample movements in Figure 13 and Figure 14, respectively. Note that these axes are relative and rotate along with the limb.

Experiments were designed to demonstrate the application and evaluation of the three DTW and four CNN classification models developed in this study. Motion capture data was collected from 15 martial artists of varying experience. 18 classes of boxing punches and 24 classes taekwondo kicks (6 kicks for each leg for shadow and bag strikes) were collected as consistent with the entire range of movements for each sport. The classification models were given no a priori information on sensor placement or left/right limb to make the systems robust to using either sensor on either limb without polarization. Moreover, the models were not presented any a priori movement features. To our knowledge, no previous system has classified this wide range of movement and no existing classification model has demonstrated generalizability to both sports [6].

**Figure 15:**
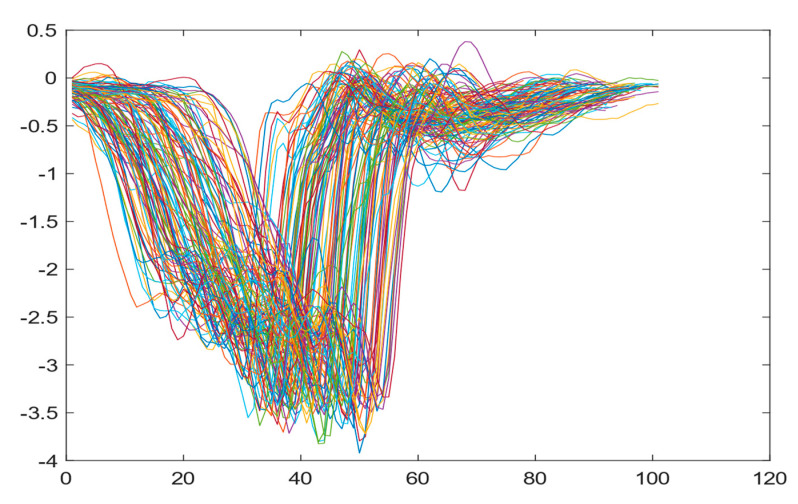
Superimposed strikes of the boxing Right Hand Shadow Hook ensemble acquired from the x-axis of the accelerometer.

**Figure 16:**
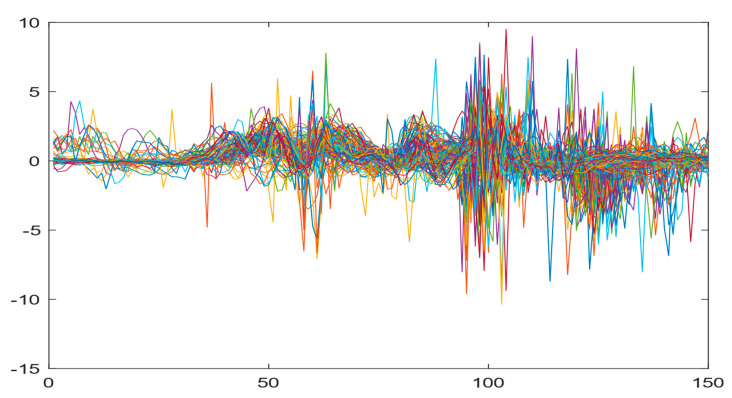
Superimposed strikes of the taekwondo Right Axe Contact Kick ensemble acquired from the x-axis of the accelerometer.

Boxing punches were acquired by placing the IMU on the wrists of each martial artist for 6 different punch classes during shadow boxing, punching a heavy bag, and with a trainer holing pads (2880 strikes, 18 classes). For taekwondo, an IMU was placed on the ankle of each martial artist executing kicking motions (2880 strikes, 24 classes). The signals were segmented using a signal-energy based algorithm [52] to locate the start and end-points of the strikes. The boxing punch classes, and taekwondo kick classes are listed in Table 1 and Table 2, respectively. Note the right- and left-hand classes will be switched for left-handed (Southpaw) boxers. Table 3 shows examples of superimposed ensembles of boxing and taekwondo strikes. For clarity, Figure 15 and Figure 16 show enlarged versions of the ensembles of one boxing and one taekwondo strike extracted from Table 3, respectively. From the blur in each figure, it is clear that signal peaks and valleys within each class are not aligned. Such nonlinear variations are typical in other boxing and taekwondo strike classes (and other human movements).

Given that the number of sensors G is 2, the number of axes $m_{g}$ in each sensor is 3, and the total number of axes $M_{g}$ is 6, the 4 input models are characterized by the following:

- VI: 6 vectors of dimension $\left(3\times n_{gm}\right)$, where $n_{gm}$ is the dimension of uni-axial signal $s_{gm}$.
- LMI: 2 matrices of dimension $\left(3\times n_{g}\right)$ , where $n_{g}$ is the normalized duration of the uni-axial signals of sensor $S_{g}$.
- GMI: A (6×N) matrix, where N is the normalized duration of all $M_{g}=6$ uni-axial signals.
- GCI: A (3×N×2)cuboid, where N is the normalized duration of all $M_{g}=6$ uni-axial signals.

## 7. System Training and Convergence

Each data set was divided randomly into a training set and a test set containing approximately 80% and 20% of the strikes, respectively. The average classification accuracy for the test set was determined. The random partitioning into training and test sets was repeated 100 times, with classification accuracies across repetitions averaged to obtain a final estimate of the classification accuracy.

For the DTW classifiers, the reference templates for the strike classes were determined by averaging the signals in their respective training sets. The CNN classifiers were initialized with a different set of random weights for each random partitioning of the data sets. Consequently, the final classification accuracy was obtained by averaging the results of 100 different CNNs. In order to keep the comparisons fair, the number of convolutions, pooling, and FC layers were fixed for all experiments. Moreover, the ordering of the layers was fixed. Given that the dimensions of the data were relatively small (2 sensors, 3 axes/sensor), a deep network with a large number of convolution and pooling layers was not needed. The CNN, therefore, consisted of a convolution layer, convolution layer, pooling layer, and 2 FC layers in which the first FC layer used sigmoidal activation functions and the last FC layer used softmax activation functions. The “same” operation was used in the convolution layer and max pooling was used in the pooling layer. The number of filters were 32 and 32 in the first and second convolution layers, respectively. The filter dimensions in the first and second convolution layers were as follows: (1 × 3 × 1) and (1 × 3 × 32) for CNN-1, (3 × 3 × 1) and (3 × 3 × 32) for CNN-2, (3 × 3 × 1) and (3 × 3 × 32) for CNN-3, and (3 × 3 × 2) and (3 × 3 × 32) for CNN-4, respectively. The networks were implemented using the Keras library [53,54,55].

Training times were benchmarked for each input model and classifier. Time efficiency profiling was conducted by using the MATLAB 2021a internal profiler for DTW models and the Python 3.8.12 c Profile function for CNN models. All evaluations were conducted on a system using Windows 10 Home Edition with an Intel Core i7-6700 k 4GHz Quad Core CPU, GeForce GTX 980 Ti GPU and 32GB RAM.

## 8. Results and Analysis

**Figure 17:**
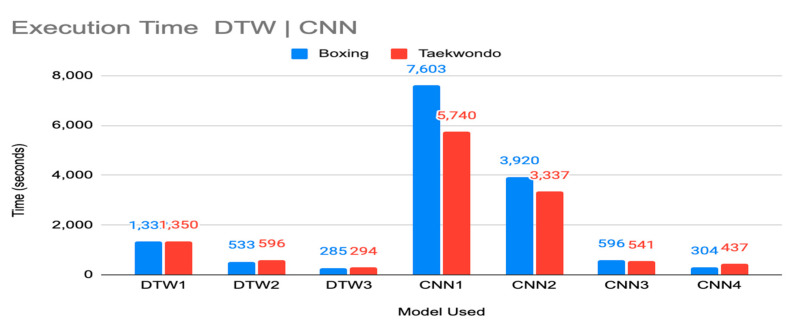
Training parameterization time for DTW and CNN models.

Figure 17 shows the total time in seconds for model parameterization. The CNN training time increase is expected given the repeated layering design as opposed to the single pass in the DTW. CNN1 and CNN2 are also setup for multiple neural networks in training, hence their increase in training times. CNN3 and CNN4, however, show parameter convergence in comparable time to DTW in the single network training. It is also interesting to note that boxing data actually took longer to train or had negligible differences to taekwondo in CNN implementations, despite being an 18-class problem versus 24-class. Boxing punches very more between the dominant and non-dominant side, but boxing must also distinguish between pad (human held and bag strike classification which could account for comparable or longer time to converge. We also note that differences between an uppercut and hook punch can include highly nonlinear variations in movement waveform.

While the training times for CNN1 and CNN2 were significant, it is also important to note that this has no impact on online use for real-time classification. Training, in particular for such large data sets will be done offline with identification in real-time occurring with trained models. Deep neural networks have well-established properties for computationally compact representations of nonlinear models enabling use online, even in time critical applications with limited computational resources (e.g., [56]). Complete identification of strikes occurs in negligible (msc) timeframes for all CNN models. DTW models are not as computationally lean for online use as they require a comparison of each data point of an incoming motion to each data point in the movement class. However, the time to execute this in real-time is still suitable for most online human-motion tracking applications. We envision training for individualized movements to be completed on cloud servers with online use parameters updated to edge devices to execute in firmware, as implemented in our commercial systems.

Outputs of the classification experiments after training are summarized in Table 4 and Table 5. Table 4 shows the results of classifying each uniaxial signal independently for both data sets. For each classifier (table row), the result of the best axis channel is shown in boldface. For example, for the uni-axial boxing DTW classifiers, the best result of 65.1% was obtained from the x-axis channel of the accelerometer. An accuracy of 65.1% may not be strong, however, in comparison, an accuracy of only 5.6% can be expected through the random classification for an 18-class problem. The classification accuracies of the seven fusion classifiers are shown in Table 5 for both data sets. The best result for each data set is shown in boldface. Note that for each classifier type and data set, the worst fusion result in Table 5 is much better than the best uni-axial result in Table 4. This clearly demonstrates the merits in fusing information from multiple sensors and axes. Also note that in Table 4 and Table 5, the accuracies of the CNN classifiers are much higher than those of the DTW classifiers.

The fact that the CNN classifiers performed better than the DTW classifiers is quite unexpected for the following reasons: (a) unlike DTW classifiers, CNNs do not readily appear to be a good choice for classifying signals that are not naturally in an 2-d or multidimensional array formats, and (b) unlike the design of DTW classifiers, the design of CNN classifier do not typically focus on addressing the non-linear variations problem. From the results, it is interesting to note the following:

(a) The CNN classifiers performed well in spite of the fact that the uni-axial strike signals typically experience non-linear variations as seen in Table 3 and in Figure 15 and Figure 16. The reason for this performance can be explained by noting that features are detected locally and not globally. Consequently, the local features tend to be invariant to latency shifts. Moreover, the local features are unaffected in the segments that do not experience non-linear variations. The trial-to-trial variations of the signals within each training set can be regarded as a natural form of “data augmentation” which is a technique commonly used to artificially increase the diversity in the training set without having to collect additional data. The CNN classifiers are capable of learning the typical variations in the signals by presenting the network with representative signals during training.
(b) Using the same input data, the CNN implementations using the four input models extracted different types of local features for classification. The CNN classifiers, therefore, offer many choices of local features which can be selected depending on the type of coupling assumed or desired between the intra and inter-sensor outputs. For example, if the uni-axial outputs of all sensors are assumed independent, CNNs using the VI model can be selected. CNNs using the LMI model can be selected if the channels in a sensor carry complementary information for determining the output class. If complementary information is shared across all sensors, CNNs using the GCI model will be an effective choice. The manner in which the inputs are fused can take other factors into account, for example, the geographical locations (co-located or dispersed) of the sensors. The sensor outputs can also be fused in other ways. For example, the x-axis channels of all sensor can be combined into an matrix. The y-axis and z-axis channels can be combined in a similar manner. The intra and inter-sensor coupling assumptions can, therefore, be used to choose a particular classification model for a given problem.
(c) It is unlikely that the performance of DTW classifiers can be improved by increasing the size of the training set because the template, which is the training set average, will change only marginally after a certain point and this marginal change will have little effect on the performance. Contrarily, CNN classifiers have the potential to improve performance by extracting more complex features by increasing the network depth and training data. Furthermore, by increasing the network depth and training data, CNNs are capable of accurately classifying a larger number of classes, whereas, the performance of traditional classifiers such as DTW classifiers will tend to drop as the number of classes increase.
(d) It is interesting to compare the performances of the one, two, and three-dimensional classifiers resulting from the VI, LMI & GMI, and GCI input models, respectively. By comparing the results for the DTW classifiers in Table 5, it is first noted that the classification accuracies vary marginally for all DTW classifiers across both sets of data. The best results for the boxing and taekwondo data were obtained by the 2-dimensional DTW-2 classifiers. The classification accuracies also varied marginally across the CNN classifiers for both data sets. The best results were obtained by the 2-dimensional CNN-3 and CNN-4 classifiers for the boxing and taekwondo data, respectively.

It is also worth noting that CNNs offer particularly intriguing potential for widespread use in commercial wearables due to low computational expense of online use. A cloud-based training system working in conjunction with an embedded wearable would enable real-time training feedback coupled with updates and adaptation as movements change with time.

Finally, it should be stressed the results presented here are designed to demonstrate the capacity of the input modelling and classification approaches in the most challenging of circumstances. Table 4 and Table 5 show output for the maximum number of classes with **zero** knowledge of movement and the broadest class of athletes. While the accuracies by themselves are enough for commercial use, further improvements are easily possible in practice. The eighteen-class boxing data, for example, yielded fusion classification accuracies of 95% + for CNN-3 with a subset (1/3) of the boxers who were not beginners. Furthermore, it is unlikely that boxers or martial artists even with simple training will mix striking a heavy bag, shadow boxing, or pad striking in the same round. Such measures will virtually eliminate misclassification such that the only errors are erratic strikes from the user that do not fit any strike model.

## 9. Translation for Mass Market Athletic Training

**Figure 18:**
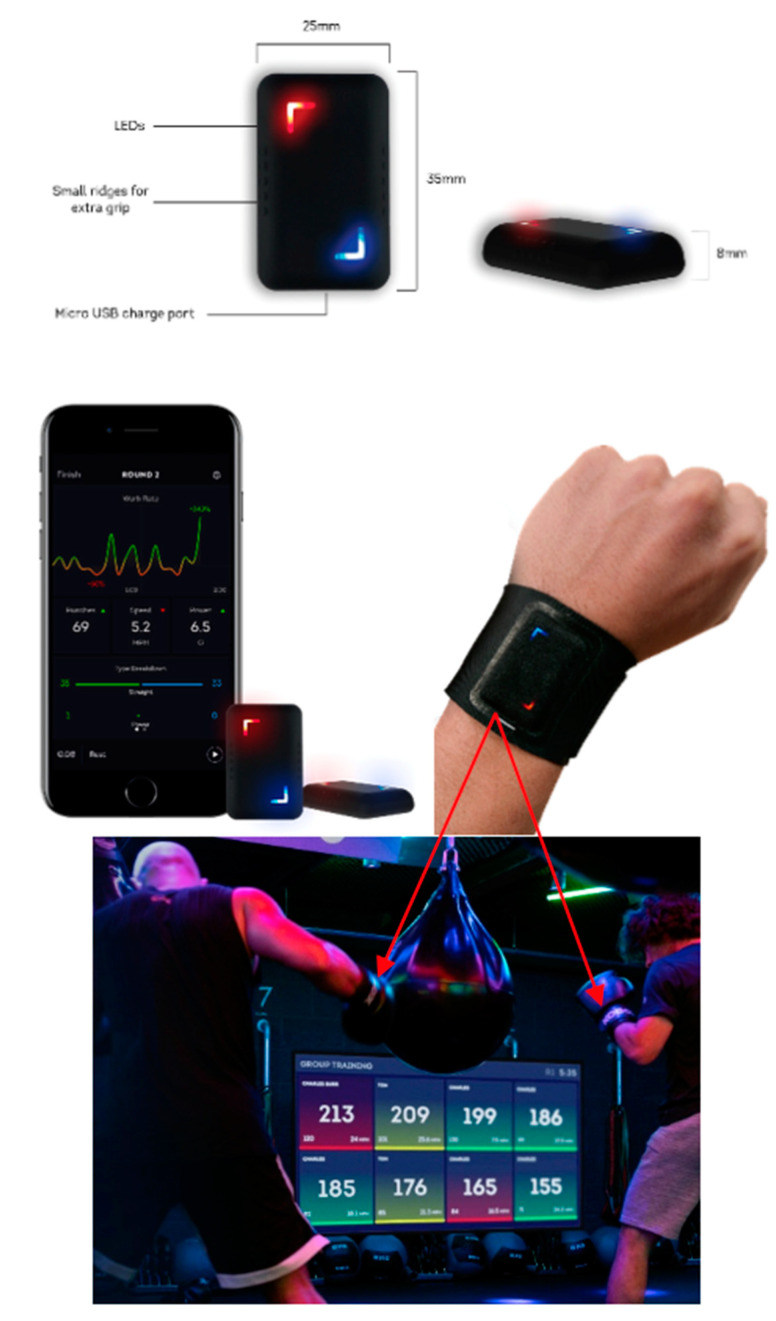
***Corner*** Boxing System: Sensor with dimensions (**top**), sensor, phone app and wrist attachment (**middle**), and sample group training session with data from several users compiled and displayed simultaneously (**bottom**).

The research executed in this investigation has led to the design, fabrication, and commercial translation of a complete IoT sensor system for smart boxing. Our design reflects the evolution of wearables from a ‘device’ to a ‘systems’ perspective [9], and consists of original sensors, embedded code, apps for use with a smart phone for data collection, and cloud computing for data storage and visualization. The system, shown in Figure 18, has been released as a commercial product by Corner Wearables based in Manchester, UK. It was first trialed with the boxing team at Imperial College London and subsequently expanded into a full product for sale worldwide. The integrated system consists of a small sensor in the boxer’s hand wraps that fits under boxing gloves. All code for punch identification is embedded onboard with a microcontroller to detect and classify movement history, which is sent via Bluetooth to a smart device for display and storage through an app on a smart phone. The embedded code performs all pattern classification hence transmission is only necessary for statistics saving the need to send raw data over Bluetooth. The first-generation commercial system tracks 6 classes of punches (dominant hand–cross, hook, uppercut, non-dominant hand–jab, hook, uppercut). Subsequent releases will classify the full 18-class problem outlined in Section 6 using the full deep learning architecture outlined in this investigation. Corner, featured in IEEE Spectrum [57], is the first ever smart boxing tracker which does not need polarized (left-right specified) sensors. It was recently assessed in a boxing study as a part of this special issue of Sensors [58] as having the capacity to track both beginners and experienced boxers, though beginner punches are less consistent due to immaturity of technique. Thousands of devices are currently in use, providing an intriguing database for analysis in future work. The commercial system has also been used in live boxing matches, including the World Series of Boxing, to provide real-time statistics to spectators, judges, and trainers to evaluate match performance.

## 10. Conclusions

The goal of this investigation was to develop models to classify human movement by fusing information from ensembles of wearable multi-axial inertial sensors. The specific contributions resulting from the investigation include: (a) the introduction of four multi-sensor multi-axial input models that can be used in conjunction with diverse classifiers, (b) demonstrating the use of the input models to develop three DTW and four CNN fusion-based classifier models that do not require a set of predetermined hand-engineered features, (c) testing the validity of the classifier on boxing and taekwondo sport data, (d) demonstrating the merits of multi-axial fusion by showing the that the worst fusion classifiers outperform the best uniaxial classifiers, (e) demonstrating that high classification accuracies can be obtained with the CNN fusion classifiers on signals that experience large non-linear variations and on signals belonging to a large number of classes, (f) demonstrating the surprising result that the CNN fusion classifiers outperform the DTW classifiers, (g) explaining the ability of the CNN classifiers to extract local features which depend on the type of coupling assumed or desired between the intra and inter-sensor outputs, and (h) noting that CNN classifiers have the potential to improve performance and handle a larger number of classes through both training and network scaling.

To our knowledge this is the first set of models demonstrated on this large a problem class in either activity [7] and the first generalized non-feature specific classification over multiple movement ranges. Also noteworthy is that due to the generalized formulations, the classifiers can be easily adapted to classify multi-dimensional signals of multiple sensors in various other applications.

Future work involves refining the system for exact learning of individual users for performance assessment, analyzing time series data from training of large groups of athletes, and implementation for live performance streaming in professional fights to enhance spectator experience support of fight scoring. As a completely feature-blind generic classification strategy, translation is also underway in other sports (e.g., tennis) as well as in wearables for telemedicine in neural motor dysfunction conditions such as stroke and Parkinson’s Disease [59,60]. We believe these results provide a foundation for a new set of human movement classification paradigms based on fusion and deep learning.

## Figures and Tables

**Table 1:** Boxing Strikes: 18 classes.

Hand	Shadow Boxing	Heavy Bag	Pads Strike
**Right**	Cross	Cross	Cross
	Hook	Hook	Hook
	Upper cut	Upper cut	Upper cut
**Left**	Jab	Jab	Jab
	Hook	Hook	Hook
	Upper Cut	Upper Cut	Upper Cut

**Table 2:** Taekwondo Kicks: 24 classes (12 shadow, 12 bag).

Kick Type (Shadow)		Kick Type (Heavy Bag)	
**Right Leg**	**Left Leg**	**Right Leg**	**Left Leg**
Turn Kick	Turn Kick	Turn Kick	Turn Kick
Axe Kick	Axe Kick	Axe Kick	Axe Kick
Front Kick	Front Kick	Front Kick	Front Kick
Back Kick	Back Kick	Back Kick	Back Kick
Side Kick	Side Kick	Side Kick	Side Kick
Reverse Hook Kick	Reverse Hook Kick	Reverse Hook Kick	Reverse Hook Kick

**Table 3:** Boxing and taekwondo strikes superimposed.

Boxing Strikes			
**Accelerometer**	**x-axis**	**y-axis**	**z-axis**
**Right Hand** **Shadow Hook**	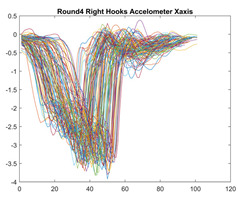	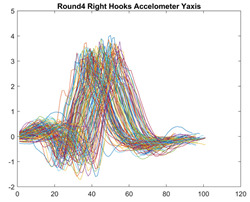	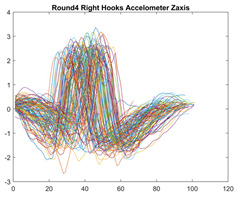
**Right Hand** **Bag Hook**	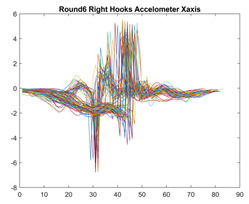	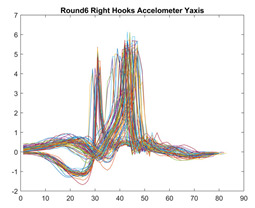	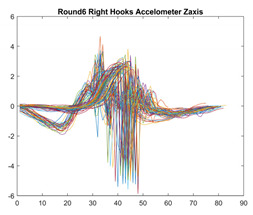
**Gyroscope**	**x-axis**	**y-axis**	**z-axis**
**Right Hand** **Shadow Hook**	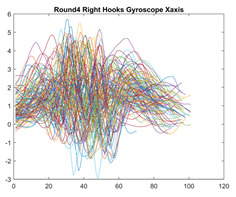	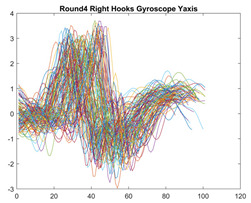	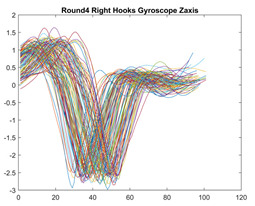
**Right Hand** **Bag Hook**	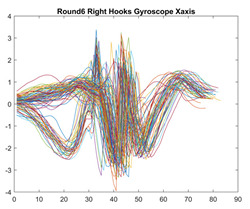	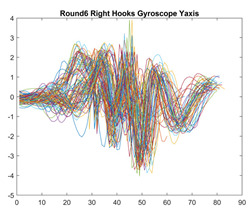	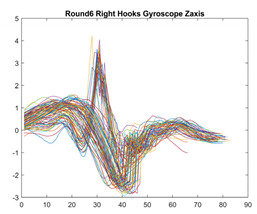
**Taekwondo Strikes**			
**Accelerometer**	**x-axis**	**y-axis**	**z-axis**
**Right Axe** **Contact Kick**	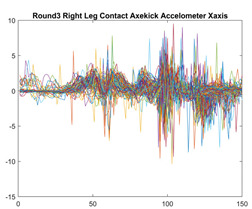	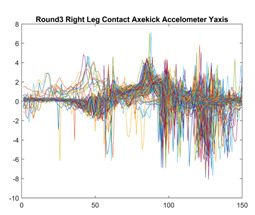	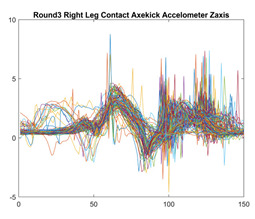
**Right Back** **Contact Kick**	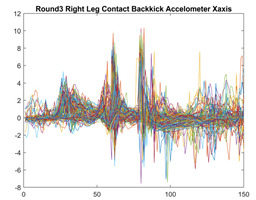	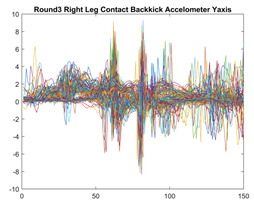	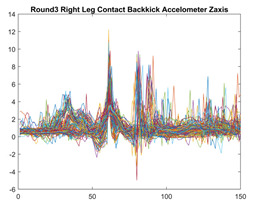
**Gyroscope**	**x-axis**	**y-axis**	**z-axis**
**Right Axe** **Contact Kick**	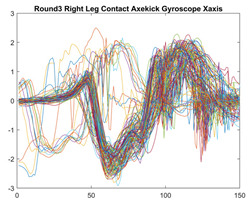	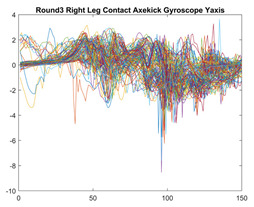	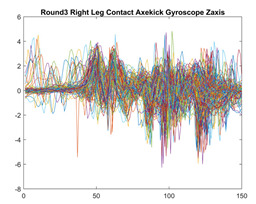
**Right Back** **Contact Kick**	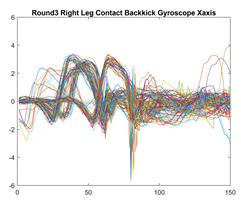	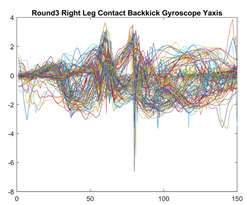	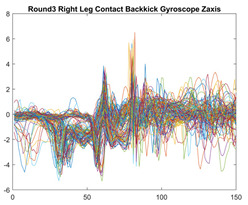

**Table 4:** Uniaxial classification accuracies (%).

		Accelerometer Axes			Gyroscope Axes		
Sport	Classifier	X	Y	Z	X	Y	Z
**Boxing**	**DTW**	**65.1**	53.1	59.4	58.1	57.9	59.4
	**CNN**	75.8	71.2	70.8	70.4	67.6	**77.1**
**Taekwondo**	**DTW**	**52.3**	46	36.4	41.2	49.7	36.4
	**CNN**	**81.7**	75.1	70.3	75.8	81.1	77.1

**Table 5:** Fusion classification accuracies (%).

Classifier	Boxing (18 Class)	Taekwondo (24 Class)
**DTW-1**	77.42	64.00
**DTW-2**	80.43	69.32
**DTW-3**	79.59	68.69
**CNN-1**	87.21	86.89
**CNN-2**	89.70	88.02
**CNN-3**	**92.08**	88.14
**CNN-4**	91.26	**88.70**

## Data Availability

Please contact corresponding authors for data availability. Ethics permission does not explicitly give permission for public release of human performance data at the time of publication.
